# Development of High-Density Genetic Linkage Maps and Identification of Loci for Chestnut Gall Wasp Resistance in *Castanea* spp.

**DOI:** 10.3390/plants9081048

**Published:** 2020-08-18

**Authors:** Daniela Torello Marinoni, Sogo Nishio, Nadia Valentini, Kenta Shirasawa, Alberto Acquadro, Ezio Portis, Alberto Alma, Aziz Akkak, Vera Pavese, Emile Cavalet-Giorsa, Roberto Botta

**Affiliations:** 1 Dipartimento di Scienze Agrarie, Forestali e Alimentari—DISAFA, Università degli Studi di Torino, Largo Paolo Braccini 2, Grugliasco, 10095 Torino, Italy; nadia.valentini@unito.it (N.V.); alberto.acquadro@unito.it (A.A.); ezio.portis@unito.it (E.P.); alberto.alma@unito.it (A.A.); vera.pavese@unito.it (V.P.); emile.cavalet_giorsa@edu.unito.it (E.C.-G.); roberto.botta@unito.it (R.B.); 2 Institute of Fruit Tree and Tea Science, NARO, 2-1 Fujimoto, Tsukuba, Ibaraki 305-8605, Japan; nishios@affrc.go.jp; 3 Kazusa DNA Research Institute, 2-6-7 Kazusa-Kamatari, Kisarazu, Chiba 292-0818, Japan; shirasaw@kazusa.or.jp; 4 Dipartimento di Scienze Agrarie, degli Alimenti e dell’Ambiente, Università degli Studi di Foggia, Via Napoli 25, 71121 Foggia, Italy; aziz.akkak@unifg.it

**Keywords:** breeding, chestnut, ddRAD-seq, *Dryocosmus kuriphilus* Yasumatsu, SSR

## Abstract

*Castanea sativa* is an important multipurpose species in Europe for nut and timber production as well as for its role in the landscape and in the forest ecosystem. This species has low tolerance to chestnut gall wasp (*Dryocosmus kuriphilus* Yasumatsu), which is a pest that was accidentally introduced into Europe in early 2000 and devastated forest and orchard trees. Resistance to the gall wasp was found in the hybrid cultivar ‘Bouche de Bétizac’ (*C. sativa* × *C. crenata*) and studied by developing genetic linkage maps using a population derived from a cross between ‘Bouche de Bétizac’ and the susceptible cultivar ‘Madonna’ (*C. sativa*). The high-density genetic maps were constructed using double-digest restriction site-associated DNA-seq and simple sequence repeat markers. The map of ‘Bouche de Bétizac’ consisted of 1459 loci and spanned 809.6 cM; the map of ‘Madonna’ consisted of 1089 loci and spanned 753.3 cM. In both maps, 12 linkage groups were identified. A single major QTL was recognized on the ‘Bouche de Bétizac’ map, explaining up to 67–69% of the phenotypic variance of the resistance trait (*Rdk1*). The *Rdk1* quantitative trait loci (QTL) region included 11 scaffolds and two candidate genes putatively involved in the resistance response were identified. This study will contribute to *C. sativa* breeding programs and to the study of *Rdk1* genes.

## 1. Introduction

Chestnut belongs to the genus *Castanea*, in the Fagaceae family, which includes *Quercus, Fagus,* and *Castanopsis.* There are four major species in the genus *Castanea:* European chestnut (*C. sativa* Mill.), Japanese chestnut (*C. crenata* Sieb. et Zucc.), Chinese chestnut (*C. mollissima* Bl.), and American chestnut (*C. dentata* Borkh.). *C. sativa* is distributed along the Mediterranean basin and Asia Minor, and it is a multipurpose species not only used for nut and wood production, but also for its contribution to the landscape in mountainous areas. This species has very good nut quality, especially the ‘Marrone’ type, which is known for the fine taste and the easy-to-remove pellicle [1]. However, this species is susceptible to two main diseases, ink disease (*Phytophthora cinnamomi* Rands) and canker blight (*Cryphonectria parasitica* Murr.) [2]. In addition, most of the *C. sativa* cultivars are susceptible to chestnut gall wasp (*Dryocosmus kuriphilus* Yasumatsu).

Interspecific hybridizations have been carried out to overcome the weak points of each chestnut species. In Europe, interspecific crosses between *C. sativa* and *C. crenata* were carried out to introduce resistance genes to ink disease, canker blight, and chestnut gall wasp [2,3,4,5]. In the USA, backcross breeding was aimed at introducing blight resistance from *C. mollissima* into *C. dentata* [6]. Moreover, *C. mollissima* accessions were introduced in Japanese chestnut breeding programs to improve the ease of pellicle removal [7]. As these papers show, interspecific hybridization is important for chestnut breeding strategies. Therefore, constructing genetic linkage maps and accumulating genetic information among chestnut species is essential for chestnut breeding programs.

The chestnut gall wasp was first introduced from China into Japan in the 1940s and spread throughout Japan in the 1960s. *C. crenata* resistant cultivars, ‘Tanzawa’, ‘Tsukuba’, and ‘Ishizuchi’ were released by a public breeding program in 1959–1968. Initially, these cultivars showed total resistance to gall wasp. However, eventually, the presence of galls was found also in these cultivars, due to the appearance of new ecotypes of the insect [8]. In 1982, the parasitoid wasp *Torymus sinensis* Kamijo (Hymenoptera: Torymidae) was released, and a rapid decrease of the infestation was obtained [9]. To date, the control of *D. kuriphilus* by *T. sinensis* has been successful in Japan.

The chestnut gall wasp was accidentally introduced into Italy and first reported in 2002. It quickly spread to all Italian regions and later into the surrounding countries [10], causing a remarkable decrease of production (−60% in 2014 in Italy). Studies on biological control aimed at introducing the parasitoid wasp *T. sinensis* and at the genetic improvement for resistance to the cynipid were promptly started to solve the problem. The susceptibility to the chestnut gall wasp was evaluated in *C. sativa* and hybrid cultivars [11]. Out of 62 cultivars, 2 *C. sativa*, 1 *C. crenata*, and 4 hybrids between *C. sativa* and *C. crenata* showed total resistance. The resistance of the hybrid cultivar ‘Bouche de Bétizac’ was extensively studied and was found to have a simple Mendelian inheritance [3]. It was hypothesized that the mechanism of resistance involves a hypersensitive reaction in the buds [12]. The presence of H_2_O_2_ and the expression of a germin-like protein gene involved in the production of reactive oxygen compounds were revealed in infested buds of ‘Bouche de Bétizac’ at budburst.

Several genetic linkage maps have been assembled for *Castanea* accessions. A map of *C. dentata* × *C. mollissima* was first constructed using random amplified polymorphic DNAs (RAPDs) allowing the detection of molecular markers associated with blight resistance [13]. Subsequently, *C. sativa* maps were built using intraspecific cross [14,15,16]. In 2013, the whole genome sequence of *C. mollissima* was released [17], consisting of 724.0 Mb in 41,260 scaffolds (N50, 39.6 Kb) with 91.2% coverage of estimated genome size (794 Mb). In the same year, a highly informative genetic map of *C. mollissima* was constructed, including 329 simple sequence repeats (SSRs) and 1064 single nucleotide polymorphisms (SNPs) markers using an expressed sequence tag database created by next-generation sequencing [18]. This consensus map consisted of 12 linkage groups ranging from 50.6 to 90.4 cM and encompassed 742.3 cM with an average distance of 0.64 cM between each pair of loci. More recent maps of *C. sativa* and *C. crenata* were constructed and anchored to the consensus map by Kubisiak et al. [18] using SNPs and anchor SSRs [4,19].

Some molecular markers associated with important agronomic traits were developed in the genus *Castanea*. The blight resistance genes of *C. mollissima* were mapped and introgressed by backcrossing into *C. dentata* [13,18]. The molecular markers associated with ease of pellicle removal were developed and applied in *C. crenata* breeding programs [19]. The quantitative trait loci (QTL) associated with agronomic traits including nut weight and pericarp splitting were identified from intraspecific crosses of *C. crenata* [20]. QTLs for adaptive traits, such as time of budburst, growth, and carbon isotope discrimination were identified in *C. sativa* [21]. In addition, QTLs for resistance to *P. cinnamomi* were identified in an interspecific cross progeny from *C. sativa* and *C. crenata*. However, molecular markers associated with ‘resistance to *D. kuriphilus’* have not been identified yet.

The genotyping by sequencing (GBS) method [22] has illustrated a cost-effective way to identify thousands of polymorphic markers. This method is based on the construction of a library based on reducing genome complexity using restriction enzymes, to ensure sufficient read depth for polymorphism discovery. Double-digest restriction site-associated DNA-Seq (ddRAD-Seq) is a modified GBS approach that involves a two-enzyme double digestion to reduce cost and time to prepare the sequencing libraries. After the double digestion, a precise size selection is applied to exclude too short and too long fragments, resulting in greater flexibility and robustness in region recovery [23]. In silico prediction prior to actual analysis contributes to optimization of the experimental conditions for ddRAD-Seq, e.g., choices of enzymes and plant materials [24]. As the cost of next-generation sequencing (NGS) has dramatically decreased [25], more and more genetic studies involved in genetic mapping, genome-wide association mapping, and population genetics have applied the ddRAD-Seq methods [24,26,27,28,29].

In this study, we used a progeny derived from the cross between the hybrid cultivar ‘Bouche de Bétizac’ (*C. sativa* × *C. crenata*, hereafter called Bouche) and *C. sativa* cultivar ‘Madonna’ (hereafter called Madonna). This population was obtained on the basis of a BC_1_ strategy aimed at introducing preferable genes from *C. crenata* to *C. sativa.* These two species have considerable sequence divergence; therefore, there is a great potential to identify many SNPs by ddRAD-Seq. The objective of this study is to construct high-density genetic linkage maps using ddRAD-Seq and to develop molecular markers associated with resistance to chestnut gall wasp to be used in chestnut breeding programs.

## 2. Results

### 2.1. ddRAD and SSR Genotyping

A total of 889.1 million (M) reads was obtained from the F_1_ seedlings and their parents by Illumina HiSeq 4000 (4.8 M reads on average). After trimming low-quality data and adapter sequences, 90.9% of 808.3 M high-quality reads were successfully mapped onto the *C. mollissima* reference genome while detecting 27,315 SNP candidates. The percentages of the mapped reads were 89.3% and 86.4% for Bouche and Madonna, respectively. After selecting SNP loci using the criteria of VCFtools described in the “Materials and Methods” section, we obtained 5451 SNPs that were heterozygous in Bouche and homozygous in Madonna, 3348 SNPs that were homozygous in Bouche and heterozygous in Madonna, and 1930 SNPs that were heterozygous in both parents. Only SNP markers segregating in either one of the parents were retained for the genetic map construction of the parent cultivars. Then, we discarded the SNPs that showed highly significant distortion from the expected 1:1 ratio and selected only one representative SNP per scaffold, resulting in 2217 and 1328 SNPs retained for Bouche and Madonna genetic linkage maps construction, respectively. We also analyzed the segregation pattern of 119 and 85 segregating SSR loci for each parent (Table S1).

### 2.2. Genetic Linkage Maps

**Figure 1:**
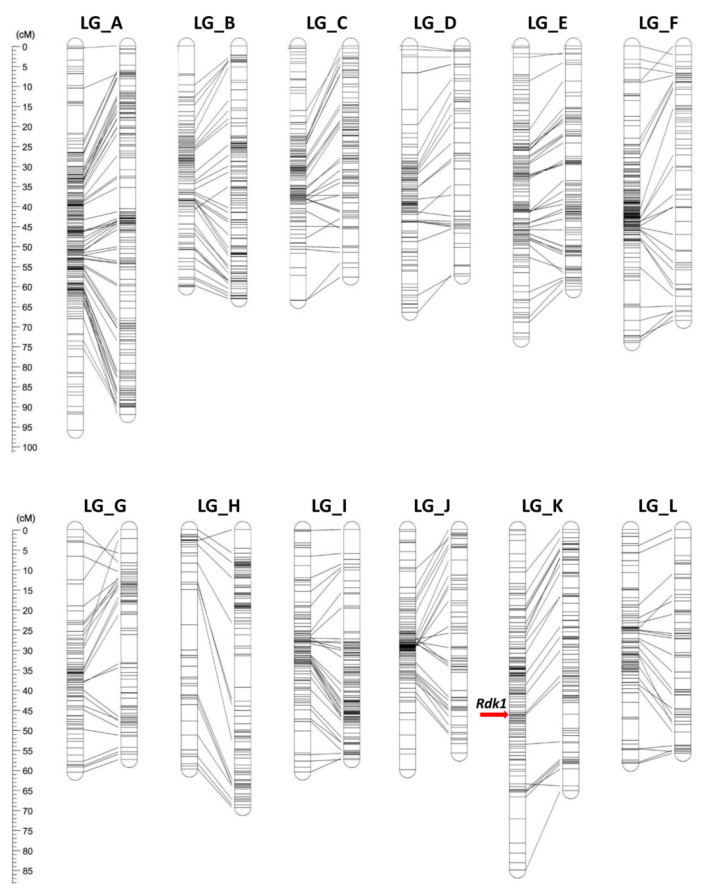
Genetic linkage maps of *Castanea* spp. ‘Bouche de Bétizac’ (Bouche) and ‘Madonna’ (Madonna) cultivars. Linkage groups (LGs) of Bouche (female parent, LGs on the left) and Madonna (male parent, LGs on the right) were aligned using markers selected on common scaffolds. The linkage group names were assigned according to the *C. mollissima* genetic consensus map [18]. The left rulers express the length of the LGs in cM. A red arrow indicates the mapping position of the quantitative trait loci (QTL) region of 1.04 cM linked to the trait of resistance to *Dryocosmus kuriphilus* (*Rdk1* QTL region).

Genetic linkage maps of Bouche and Madonna were constructed using the pseudo-test cross strategy (Figure 1). A total of 862 and 308 co-segregating markers located at identical loci were excluded for Bouche and Madonna, respectively, to improve calculation efficiency. We identified 12 linkage groups (named with letters from A to L) in both parent maps, corresponding to the haploid chromosome number of the species. Maps were successfully aligned to the previously available *C. mollissima* consensus map by using anchor SSRs (Figure S1). The developed linkage groups showed a one-to-one correspondence with the groups in the consensus map. The order of the SSRs in each group was very similar in both maps.

The genetic linkage map of Bouche contained 1459 loci, including 119 SSRs and 1340 SNPs (Table 1). The total length of the map was 809.6 cM with an average interval of 0.55 cM between loci. The number of mapped scaffolds was 2202 and the total length of scaffolds was 127.2 Mb, which was equivalent to 16.0% of the estimated genome size (794 Mb). A region characterized by strong segregation distortion was identified on Bouche_H, influencing the number of markers mapped on this linkage group (LG) (47), which was smaller than the number on the other LGs (on average 121.7), since we discarded the SNPs showing significant segregation distortion (*p* < 0.01). There were no gaps >10 cM on any LGs. However, there were many markers mapping in the middle part of the LGs, while fewer markers mapped to the distal ends of LGs (Figure 1). For example, out of 119 loci mapped on Bouche_C, 79 loci (66.3%) mapped between 20 and 40 cM (31.6% of length of Bouche_C).

The genetic linkage map of Madonna contained 1089 loci, including 85 SSRs and 1004 SNPs (Table 1). The total length of the map was 753.3 cM with an average interval of 0.69 cM between loci. The number of mapped scaffolds was 1313 and the total length of scaffolds was 75.2 Mb. A strong segregation distortion region was identified on Madonna_D and Madonna_J. Similarly to the map of Bouche, the number of mapped loci on these LGs were smaller than those on other LGs (49 and 60 versus the average number of 90.8). Compared with the map of Bouche, the markers were more uniformly distributed along the LGs (Figure 1).

### 2.3. Phenotypic Distribution of Dryocosmus Kuriphilus Susceptibility

The response to *D. kuriphilus* in F_1_ seedlings and their parents was evaluated under controlled conditions. While Bouche showed total resistance (no galls at all), Madonna showed a medium-high level of susceptibility (with an average of 0.56 galls/bud) [11]. Concerning the F_1_ seedlings, the number of galls per plant was zero for the resistant genotypes, but ranged in the first year of trial from 5 to 59 and for the second year of trial from 17 to 132 for the susceptible individuals, with a level of infestation ranging between 0.17 and 2.21 galls/bud for the first year and between 0.25 and 1.13 galls/bud for the second year. Out of the 139 seedlings evaluated, 63 individuals were classified as resistant, while 76 were classified as susceptible, indicating a simple Mendelian segregation, χ^2^ (1:1) = 1.22 (α = 0.05) (Table S2).

### 2.4. QTL Determining Resistance to Dryocosmus Kuriphilus (Rdk1 QTL Region)

An independent QTL analysis was performed for each season (Table 2). The initial identification procedure, by means of the simple interval mapping (SIM) procedure, highlighted two genomic regions influencing resistance to *D. kuriphilus,* both of them on the Bouche map, on LG_D and LG_K. The same QTL regions were identified in both seasons and were thus taken forward into the multiple QTL mapping (MQM) procedure. Only the QTL region on LG_K was confirmed as being above the genome-wide logarithm of odds (LOD) thresholds (GW), which was determined by the permutation test at *p* ≤ 0.05. Then, a single major QTL (*RdK1*) was identified. It was linked to the marker sca03566_22494 and its co-segregating SNPs sca07739_7819, sca10655_2698, and sca00261_32530, and explained 67.2% to 69.4% of the phenotypic variance (PVE) in both seasons. Further five marker loci (and two co-segregating SNP) were found in the 1.04 cM QTL region, which were associated with a LOD greater than the genome-wide threshold. Table 2 shows the properties of the *Rdk1* QTL region identified: scaffold location on the genetic map, LOD value, and proportion of PVE at the QTL peak identified. The nearest SSR locus was 4_145, and it was linked to the *Rdk1* QTL region with a map distance of 4.4 cM (Figure S1). At the other side, EMCs22 and CmSI0611 were linked to *Rdk1* with a map distance of 6.7 cM and 9.0 cM, respectively. We checked the pattern of allele transmission of 4_145 and CmSI0611 using ‘Bouche Rouge’ and CA04, the parents of Bouche, and confirmed that the alleles linked to the resistance gene were derived from the Japanese parent CA04.

### 2.5. Identification of Candidate Genes Within the Rdk1 QTL Region

A total of 26 genes were detected in the *Rdk1* QTL region (Table 3). Two genes related to pathogenesis and to hypersensitive response, with orthologous loci in *Arabidopsis thaliana,* were identified. The first gene found on scaffold 06906 corresponds to the AT1G02170.1 locus of *A. thaliana*. The locus codes for a metacaspase-1b protein, which is a main agent in the apoptosis process and in particular in the hypersensitive response. Expression data showed higher levels of expression in Bouche versus Madonna in both infested (0.79 GFOLD) and non-infested (0.58 GFOLD) condition. The second gene identified on scaffold 18444 corresponds to *Arabidopsis* locus AT3G14470.1, and it encodes for a receptor belonging to the NB-LRR family and the RPP13 subfamily [30]. In this case, levels of expression of Bouche versus Madonna were 0.16 and 0.35 GFOLD in infested and non-infested buds, respectively.

## 3. Discussion

A progeny produced by crossing a resistant hybrid of *C. sativa* × *C. crenata* and a susceptible cultivar of *C. sativa* was used to evaluate the response to gall wasp.

We constructed high-density genetic maps of Bouche and Madonna using the pseudo-test cross strategy. While the map of Madonna represents a pure *C. sativa* map, that of Bouche refers to an interspecific cross between *C. sativa* x *C. crenata*. Since recombination between *C. sativa* and *C. crenata* would occur in a gamete from Bouche, this BC_1_ population would have diverse genetic variation. The number of mapped markers was 2321 for Bouche and 1397 for Madonna. These numbers are larger in comparison with data of previous genetic maps of *Castanea*, which used SSRs and SNP GoldenGate assay [4,18,20]. The total length of the map was 809.6 cM for Bouche and 753.3 cM for Madonna. These data are higher than those found for previous genetic maps, e.g., 498.9 cM for the integrated map of *C. sativa* x *C. crenata* [4], 668.1 cM for *C. crenata* [20], and 721.1 cM for *C. mollissima* [18]. In addition, there were no gaps larger than 10 cM on both maps. Since we mapped considerable numbers of SNPs and SSRs, we were able to construct highly saturated maps without losing information. Each LG of the maps showed a one-to-one correspondence with one of the LGs in the *C. mollissima* consensus map [18] (Figure S1). The order of anchor SSRs in each LG was very similar in all maps, showing high collinearity.

There was a large difference in marker distribution between the maps of Bouche and Madonna. While markers were uniformly mapped on LGs of Madonna, in Bouche, most markers were densely distributed on the central part of LGs and fewer on the distal parts of LGs (Figure 1). This biased marker distribution was found only in the Bouche map among previously developed chestnut genetic maps [4,18,20], suggesting that the lower genome homology between *C. sativa* and *C. crenata* had affected recombination. This agrees with previous reports showing that interspecific hybridization reduces recombination and map size compared with intraspecific hybridization [32,33,34,35]. These studies, carried out on other species, revealed a reduction of recombination at the end of LGs, similarly to our results. However, the reason why the recombination increased in the central part of the LGs of Bouche is unclear. Meiotic recombination frequency varies extensively both within and between species [36]; thus, it would be difficult to explain this biased marker distribution from only one map. Further backcross genetic studies would be needed to clarify the difference in recombination frequency between central and distal part of LGs.

There are no recent studies on the resistance of *C. crenata* cultivars, while papers of the breeding period in Japan reported a major resistance source found in the cultivar ‘Ginyose’. These studies on the basis of resistance agree that more mechanisms may be responsible for the resistance or tolerance response in different genotypes. The resistance found in ‘Bouche de Bétizac’ involves a hypersensitive reaction [12], as described in ‘Ginyose’ by Shimura [8], and the *Rdk1* QTL region was mapped on LG_K of Bouche. This cultivar is an offspring of *C. sativa* ‘Bouche Rouge’ (susceptible to *D. kuriphilus*) and *C. crenata* CA04, an INRA selection (resistant to *D. kuriphilus*). Out of the F1 seedlings evaluated, the percentage of resistant and susceptible individuals suggested the presence of a monogenic resistance in Bouche in the heterozygous state. We checked the allele inheritance of the flanking SSRs 4_145 and CmSI0611 determining the parent profiles and confirmed that the allele linked to the resistance gene comes from CA04. Resistance was also ascertained in other European–Japanese hybrids, such as ‘Vignols’ and ‘Maridonne’, which share the common parent CA04 [11]. Moreover, CA04 has a parent–offspring relationship, based on 30 SSRs, with the cultivar ‘Dengrou’ (*C. crenata*), selected in Japan. This cultivar was reported to have high resistance to *D. kuriphilus* [8]. Out of the 26 genes identified in the *Rdk1* QTL region, two candidate genes were of particular interest. The gene AT1G02170.1 codes for a metacaspase-1b protein that is recognized as a main agent in the apoptosis process and, in particular, in the hypersensitive response. Coll et al. [37] found that silencing metacaspase-1b in *A. thaliana* removes the hypersensitive response induced by pathogenesis receptors. The gene expression, in bud tissues, was greater in Bouche compared with Madonna, both for infested (0.79 GFOLD) and non-infested buds (0.58 GFOLD) [31]. This observation suggests that the gene coding for metacaspase-1b is constitutively more expressed in Bouche than in Madonna. The low level of gene expression is probably involved in the lack of hypersensitive response in Madonna, as shown by Dini et al. [12]. The gene AT3G14470.1 encodes for a receptor belonging to the NB-LRR family and the RPP13 subfamily. The receptor of the RPP13 subfamily recognizes pathogen effectors through the LRR domain. The receptor is known to be involved in a hypersensitive response reducing pathogen growth [38]. Yet, since there is a low gene expression difference between Bouche and Madonna [31], this gene needs to be investigated further. A future development of the research could consider transcriptomic analyses on resistant and susceptible F1 seedlings, in order to better understand the involvement of these two genes in gall wasp resistance and to increase knowledge on the resistance response to pests.

The parasitoid *T. sinensis* was first released in 2005 in Italy and then successfully established in 8–10 years, forming a stable population, following the success in Japan [39,40]. In Japan, after the *T. sinensis* settlement in 1980s, there were three peaks in the population numbers of *D. kuriphilus,* shortly followed by increases in the population of *T. sinensis* (Moriya, personal communication). This showed that although *D. kuriphilus* has not been a significant problem for chestnut production in Japan for the last 25 years, the infestation of the pest may fluctuate depending on year and location. In Japan, most of the susceptible cultivars were replaced by resistant cultivars in the 1970s, but this was not sufficient to solve the problem, and the introduction of the parasitoid was required. The use of cultivars bearing resistance or low susceptibility to the pest, combined with the use of biological control by the natural parasitoid *T. sinensis*, has been successful in different parts of the world and has contributed to control infestations and to reduce yield losses. Nevertheless, the study of the resistance reaction and its genetic basis appears of extreme interest for the identification of genes involved in plant–insect interactions and for the development of future breeding programs. In fact, finding different sources of resistance could enable gene pyramiding, which could provide a long-term solution for pest control.

## 4. Materials and Methods

### 4.1. Plant Materials

The F_1_ population (250 F_1_ seedlings) was obtained from a cross between the hybrid cultivar ‘Bouche de Bétizac’ (*C. sativa* × *C. crenata*, hereafter called Bouche), as the female parent, and *C. sativa* cultivar ‘Madonna’ (*C. sativa*, hereafter called Madonna), as the male parent [12]. Bouche is a cultivar showing full resistance to *D. kuriphilus*, while Madonna is highly susceptible to the pest.

At the end of May, branches of Bouche were isolated using pollen-proof bags to exclude foreign wind-borne pollen. Before bagging, the branches were emasculated by clipping off the catkins to avoid potential uncontrolled pollination. At the beginning of June, pollen was collected from limbs of a selected plant of Madonna. The limbs were placed indoors in a closed room at 20 °C overnight and, the next day, pollen was collected from catkins, poured into glass vials, and stored at 4 °C until artificial pollination. The pollen of Madonna was manually applied to Bouche using a paintbrush when the female flowers were receptive. The pollen-proof bags were removed at the end of July and replaced with a bag of plastic net to mark the nuts obtained by cross pollination.

The nuts were collected in September after natural fall from the burr and kept stratified in wet peat at 4 °C. Subsequently, the seeds were sown in pots filled with a substrate composed of peat and perlite (3:1 ratio) and kept in a greenhouse until late May to early June. In June of the following year, one-year-old seedlings were transferred into the nursery to be tested for resistance/susceptibility after controlled infestation with *D. kuriphilus*.

After verifying resistance/susceptibility to chestnut gall wasp, the seedlings and three trees each of the two parent cultivars were planted in a field located at the Piemonte Regional Forest Nursery ‘Gambarello’, in Chiusa Pesio (Cuneo province) (44°30′ N; 7°68′ E; 575 m a.s.l.). The plants were trained in a free system with a spacing of 5 × 6 m, without water supply, and fertilization was supplied every year by UNISLOW 21-8-16. The cover grass was mowed and chopped during the growing season, with copper-based bactericide applied in autumn and spring, while no insecticide treatments were delivered during the trial.

A set of 139 F1 individuals of the progeny was selected for genotyping and phenotyping, and it represents the mapping population.

### 4.2. Simple Sequence Repeats Genotyping

Genomic DNA was extracted from young leaves using the CTAB method [41].

The previously developed 141 SSR loci from *C. sativa*, *C. mollissima*, *C. crenata*, *Quercus rubra,* and *Q. petraea* ([18,42,43,44,45,46,47], Akkak pers. commun.) were amplified for genotyping (Table S1).

PCR reactions were performed in a volume of 10 µL containing 1× PCR buffer, 200 µM of each dNTP, 0.2 µM of reverse primer, and 0.16 µM of 5′-labeled M13 tail, 0.04 µM of forward primer (with complementary M13 tail sequence added to 5′ end), 0.1 U of KAPA Taq DNA polymerase (KAPABIOSYSTEMS, Wilmington Massachusetts, USA), and 2.5 ng of template DNA. Amplification was performed in 35 cycles of 94 °C for 1 min, 55 °C for 1 min, and 72 °C for 2 min. Amplification products were diluted 10× and analyzed on a 3130 Genetic Analyzer (Applied Biosystems^®^, Foster City, CA, USA). The internal GeneScan^TM^ size standard 500 LIZ was included in each run. Allele sizes in the output were called using GeneMapper v. 4.0 software (Applied Biosystems^®^, Foster City, CA, USA).

### 4.3. ddRAD-Seq Library Construction and Sequencing

ddRAD-Seq library was constructed as described by Shirasawa et al. [24]. A total of 200 ng of genomic DNA for each individual was double-digested with *Pst*I and *Msp*I (FastDigest restriction enzymes; Thermo Fisher Scientific, Waltham, MA, USA), ligated to adapters using the LigaFast Rapid DNA Ligation System (Promega, Madison, WI, USA), and purified using Agencourt AMPure XP (Beckman Coulter, Brea, CA, USA) to eliminate short (<300 bp) DNA fragments. Purified DNA was diluted with H_2_O and amplified by 20 cycles of PCR with indexed primers. Amplicons were pooled and separated on a BluePippin 1.5% agarose cassette (Sage Science, Beverly, MA, USA), and fragments of 300–900 bp were purified using the QIAGEN Mini Elute Kit (Qiagen, Hilden, Germany). Then, the library was sequenced using a HiSeq4000 (Illumina, Inc., San Diego, CA, USA).

### 4.4. SNP Detection

**Figure 2:**
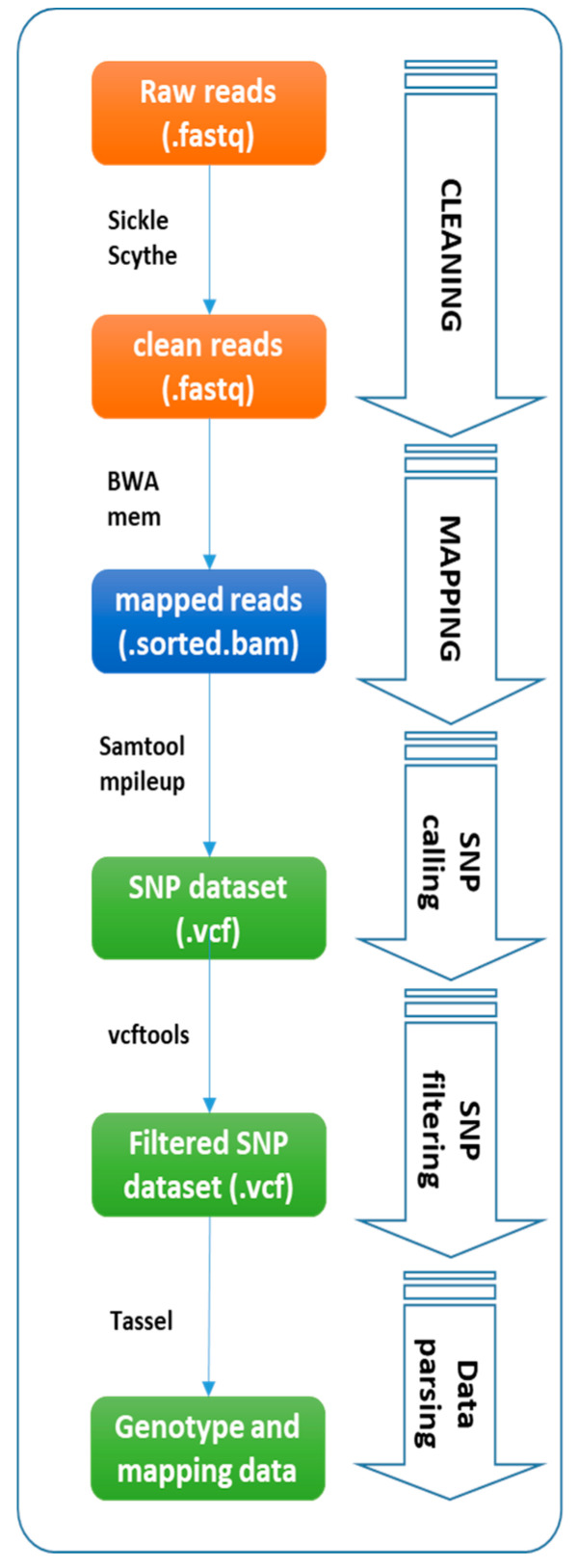
Samtools-based pipeline. The five steps of the Samtools-based pipeline for SNP and indel mining.

SNPs and indels were identified according to Acquadro et al. [48] (Table S3). Illumina reads were de-multiplexed on the basis of the Illumina TruSeq index. Raw reads were analyzed with Scythe (https://github.com/vsbuffalo/scythe) for filtering out contaminant substrings and Sickle (https://github.com/najoshi/sickle), which allows the removal of reads with poor quality ends (Q < 30). Alignment to the reference *C. mollissima* genome (Cm_v1.1_scaffolds.fasta) [17] was carried out using a BWA aligner [49] (e.g., mem command) with default parameters and avoiding multiple-mapping reads. SNP and indel mining was conducted by adopting a Samtools-based pipeline [50] as presented in Figure 2 and in File S1; in particular, the mpileup command was adopted with default parameters except for (1) minimum mapping quality (Q = 20) and filtering out multimapping events (−q >  1). SNPs and indels (hereafter called SNPs) detected from the alignments were filtered with VCFtools (version 0.1.13; parameters: –minQ 20 –max-missing-count 20 –minDP 10 –maf 0.1 –maf 0.9 –min-allele 2 –max-allele 2), as presented in Figure 2 and in File S1.

### 4.5. Genetic Linkage Map Construction

JoinMap v. 4.1 [51] was applied to develop the maps of ‘Bouche de Bétizac’ and ‘Madonna’ by adopting the pseudo-test cross mapping strategy in the BC_1_ mode [52]. We used the following marker type for the construction of separated genetic maps: (1) SNPs that were heterozygous in the maternal parent and homozygous in the paternal parent; (2) SNPs that were homozygous in the maternal parent and heterozygous in the paternal parent; and (3) SSRs that were heterozygous in the maternal or paternal parent only. SNPs showing significant segregation distortion (χ^2^ test, *p* < 0.01, d.f. = 2) were excluded. Only one marker per scaffold was selected and used for the map construction. To improve calculation performance, markers with identical genotypes were excluded using the “similarity of loci” command. For both maps, linkage groups (LGs) were established based on a threshold logarithm of odds (LOD) ratio of 8.0 with a recombination frequency of 0.45. The regression mapping algorithm was used to build the LGs, and map distances were calculated according to Kosambi’s mapping function [53]. The LG names were assigned according to the *C. mollissima* linkage maps by Kubisiak et al. [18]. The genetic maps were drawn using MapChart ver. 2.2 [54]. The distorted SSRs were marked with *, **, and ***, for which *p* = 0.05, 0.01, and 0.001, respectively.

### 4.6. Phenotypic Evaluation of Dryocosmus Kuriphilus Susceptibility

The seedlings were tested for being resistant or susceptible under controlled infestation of *D. kuriphilus* for 2 years. Starting in June, they were kept in metallic structures (‘modules’) covered by aphid-proof netting and during summer also by shading net (Figure S2). The total number of buds for each seedling was counted before releasing the insects.

*D. kuriphilus* infestation was performed under high-pressure conditions using a ratio of one female adult per 2.5 buds. The number of buds per plant ranged from 11 to 79 in the first year, and from 27 to 144 in the second year. The number of galls developed on each seedling was counted in the following summer (June–July) of each year of the infestation trial, to assign the resistance (0 galls/plant) or the susceptibility (≥1 galls/plant) status. The symptomless individuals were checked by visual assessment for another year, after planting in an open field.

### 4.7. Quantitative Trait Loci (QTL) Detection

QTL detection was performed considering independently each season and was based on the newly developed maps using both the simple interval mapping procedure (SIM) [55] and the multiple QTL mapping (MQM) [56] as implemented in MapQTL v4 software [57]. Among the markers lying within a region harboring a QTL, the one associated with the highest LOD score was used as a co-factor. For the MQM, a backwards elimination procedure was applied to select the appropriate co-factors. LOD thresholds for QTL significance were confirmed using a permutation test comprising 1000 replications, which implies a genome-wide significance level of 0.05 [58]. Only QTLs associated with a LOD greater than either the genome-wide threshold or the LG threshold were considered. The proportion of the overall phenotypic variance explained (PVE) associated with each QTL was also estimated from the MQM model.

### 4.8. Expression Analysis of Genes Within the Rdk1 QTL Region

The function and expression of genes included in the *Rdk1* QTL region were analyzed. Scaffolds transcript sequences were obtained using the *C. mollissima* genome [17]. The functional annotation of the genes in this region was based both on the annotation of the *C. mollissima* genome and the Bouche transcriptome [31]. An analysis of *A.* *thaliana* orthologues was performed in order to find genes related to pathogen response using the Uniprot database [30].

The results of differential expression genes analysis obtained by Acquadro et al. [31] were used to support functional analysis. After mapping the reads on the reference transcriptome, they identified gene expression levels using the “dif” function of the GFOLD algorithm. The analysis of the transcriptome profiles of the two cultivars, Bouche (B) and Madonna (M) was performed using four pairwise comparisons: BI versus BNI, BI versus MI, MI versus MNI and BNI versus MNI, in which “I” stands for infested and “NI” stands for non-infested.

## 5. Conclusions

Euro-Japanese F1 hybrids cultivars in Europe were obtained by INRA Bordeaux to increase the resistance of cultivated chestnuts to ink disease and canker blight. Recently, some of these cultivars showed the interesting trait of resistance to gall wasp. However, the nut organoleptic quality of the hybrid cultivars is considered much lower than that of *C. sativa* cultivars due to the lower quality of the Japanese chestnuts. Nevertheless, *C. crenata* can be seen as a major source of genes of resistance or tolerance to pests and pathogens. Once these genes are known, the acquired knowledge can be used in breeding programs. A large effect QTL, expressed across two growing seasons, was mapped on the Bouche map linkage group K and explained up to 67–69% of the phenotypic variance of the response to *D. kuriphilus*. A putative gene for a metacaspase-1b proteins was found in one of the scaffolds linked to the *Rdk1* QTL region. The high-density maps developed in this study support further genetic studies, and once a better reference genome will be available, it will allow a more in-depth exploration of the regions flanking the trait. In addition, the obtained BC1 progeny can be used to develop molecular markers for resistance to chestnut blight and ink disease as well as for other agronomic traits, including nut quality. Further analysis on progenies from different parental lines or genome-wide association (GWAS) approaches could contribute to finding more regions of interest as well as to confirm the newly identified one.

## Figures and Tables

**Table 1:** Characteristics of ‘Bouche de Bétizac’ (Bouche) and ‘Madonna’ (Madonna) linkage maps.

Linkage Group	Number of Loci	Length of Linkage Groups (cM)	Number of Markers	Average Loci Interval (cM/Loci)	Mapped Scaffolds	Total Length of Scaffolds (bp)
Bouche_A	217	95.6	335	0.44	322	20,080,944
Bouche_B	123	59.9	236	0.49	227	12,693,145
Bouche_C	119	63.3	186	0.53	179	13,168,399
Bouche_D	110	66.2	169	0.60	157	11,638,867
Bouche_E	156	72.9	223	0.47	216	9,764,018
Bouche_F	163	73.8	238	0.45	229	14,708,920
Bouche_G	86	59.6	161	0.69	150	7,498,312
Bouche_H	47	58.8	82	1.25	71	4,308,112
Bouche_I	103	59.5	172	0.58	166	8,554,610
Bouche_J	119	59.0	201	0.50	189	10,514,064
Bouche_K	131	83.6	195	0.64	182	8,501,984
Bouche_L	85	57.4	123	0.68	114	5,773,207
**Bouche total**	**1459**	**809.6**	**2321**	**0.55**	**2202**	**127,204,582**
Madonna_A	171	91.7	257	0.54	249	15,914,736
Madonna_B	118	62.9	148	0.53	143	7,642,270
Madonna_C	97	57.5	126	0.59	121	9,479,624
Madonna_D	49	57.2	57	1.17	49	3,338,321
Madonna_E	105	60.7	142	0.58	138	6,422,502
Madonna_F	66	68.3	85	1.03	78	4,119,360
Madonna_G	72	56.5	89	0.78	80	3,607,147
Madonna_H	99	68.2	119	0.69	109	6,640,418
Madonna_I	107	56.5	132	0.53	126	6,420,027
Madonna_J	60	54.9	67	0.91	59	3,508,456
Madonna_K	90	64.1	110	0.71	101	4,308,112
Madonna_L	55	54.9	65	1.00	60	3,621,485
**Madonna total**	**1089**	**753.3**	**1397**	**0.69**	**1313**	**75,022,458**

**Table 2:** Summary of the scaffolds comprised within the *Rdk1* QTL region on linkage group Bouche K. The table indicates genome-wide LOD thresholds for each season (GW) as determined by a permutation test at *p* ≤ 0.05, the markers included in the QTL region (scaffold position) and their map location (cM), the estimated LODs, and the proportions (%) of the total phenotypic variance explained (PVE) at the QTL peak (position 45.41 cM).

Scaffold	Scaffold Position (bp)	LG Position (cM)	First Season			Second Season		
			GW	LOD	PVE	GW	LOD	PVE
scaffold03566	22494	**45.41**	**3.2**	**3.55**	**67.2**	**3.3**	**3.62**	**69.4**
scaffold07739	7819	45.41						
scaffold10655	2698	45.41						
scaffold00261	32530	45.41						
scaffold07319	12557	45.60		3.40			3.55	
scaffold18444	371	45.83		3.32			3.40	
scaffold06031	22490	46.09		3.22			3.38	
scaffold04450	24357	46.38		3.22			3.38	
scaffold05894	16508	46.45		3.22			3.38	
scaffold06906	12722	46.45						
scaffold10721	6288	46.45						

**Table 3:** List of 26 candidate genes identified in the *Dryocosmus kuriphilus* (*Rdk1*) QTL resistance region of the chestnut cultivar ‘Bouche de Bétizac’. *A.* *thaliana* orthologues based both on the annotation of the *C. mollissima* genome and the Bouche transcriptome are presented. Gene expression levels using the “dif” function of GFOLD algorithm ‘Bouche de Bétizac’ (B) and ‘Madonna’ (M) in which “I” stands for infested and “NI” for non-infested, as described by Acquadro et al. [31].

Scaffold (*C. mollissima* Genome)	Name of Gene (*C. mollissima* Genome)	*A. thaliana* Orthologues (*C. mollissima* Genome)	*A. thaliana* Orthologues (Bouche Transcriptome)	MI_vs_MNI (GFOLD)	BI_vs_BNI (GFOLD)	BI_vs_MI (GFOLD)	BNI_vs_MNI (GFOLD)
scaffold00261	maker-scaffold00261-augustus-gene-0.29-mRNA-1	AT2G44900; AT3G60350.1	AT2G44900.1	0.25	0.53	0.87	0.59
scaffold00261	maker-scaffold00261-snap-gene-0.32-mRNA-1	AT2G44910.1; AT4G37790; AT2G22800.1; AT3G60390.1	AT3G60390.1	1.35	0.30	0.00	0.87
scaffold00261	maker-scaffold00261-snap-gene-0.33-mRNA-1	AT3G60370.1	AT3G60370.1	0.14	0.06	−0.20	−0.25
scaffold00261	maker-scaffold00261-augustus-gene-0.30-mRNA-1	AT5G65274.1; AT4G01710.1	-	0.71	0.00	0.14	1.51
scaffold00261	maker-scaffold00261-snap-gene-0.36-mRNA-1	AT1G05940.1	AT1G05940.1	0.00	0.35	0.62	0.10
scaffold00261	maker-scaffold00261-snap-gene-1.35-mRNA-1	AT3G59670.1	AT3G59670.1	−0.05	0.00	0.00	−0.11
scaffold00261	maker-scaffold00261-snap-gene-1.36-mRNA-1	AT4G37340.1; AT4G37360.1; AT4G37330.1; AT3G53280.1	AT4G37370.1	−1.43	0.14	0.15	−1.44
scaffold00261	maker-scaffold00261-augustus-gene-1.31	AT3G60340.1	AT3G60340.1	0.33	−0.41	−0.29	0.44
scaffold00261	augustus_masked-scaffold00261-abinit-gene-1.1-mRNA-1	AT2G13600.1; AT2G22410; AT5G19020.1	AT1G08070.1	0.00	0.00	0.23	0.19
scaffold05894	augustus_masked-scaffold05894-abinit-gene-0.0-mRNA-1	AT3G60220.1	AT3G60220.1	0.00	−0.04	0.78	0.92
scaffold03566	maker-scaffold03566-snap-gene-0.12-mRNA-1	AT2G44745.1; AT4G39410	AT2G44745.1	0.00	0.00	0.51	0.89
scaffold04450	maker-scaffold04450-augustus-gene-0.14-mRNA-1	AT2G44520.1	AT2G44520.1	0.17	0.50	0.57	0.23
scaffold04450	maker-scaffold04450-snap-gene-0.17-mRNA-1	AT2G44525.1	AT3G16000.1	0.26	0.00	−0.24	−0.23
scaffold05894	maker-scaffold05894-snap-gene-0.11-mRNA-1	AT1G02180.1	AT1G02180.1	0.00	0.00	0.00	0.22
scaffold06031	maker-scaffold06031-augustus-gene-0.16-mRNA-1	-	AT3G26330.1	0.00	0.00	0.69	0.54
scaffold06906	maker-scaffold06906-snap-gene-0.18-mRNA-1	AT2G35170.1; AT1G21920.1	AT4G17080.1	0.31	0.20	0.00	0.04
scaffold06906	maker-scaffold06906-snap-gene-0.19-mRNA-1	AT4G25110.1; AT1G02170.1	AT1G02170.1	0.00	0.01	0.79	0.58
scaffold06906	maker-scaffold06906-snap-gene-0.20-mRNA-1	AT3G10950.1; AT3G60245.1	AT3G10950.1	0.28	0.13	0.52	0.65
scaffold07319	maker-scaffold07319-augustus-gene-0.10-mRNA-1	AT2G39445.1	AT3G09080.1	0.00	0.00	0.37	0.75
scaffold07319	maker-scaffold07319-augustus-gene-0.8-mRNA-1	AT1G80770.1	AT1G80770.1	0.00	0.00	0.00	0.00
scaffold07319	maker-scaffold07319-augustus-gene-0.8-mRNA-2	AT1G80770.1	AT1G80770.1	0.00	0.00	0.00	0.00
scaffold07739	maker-scaffold07739-augustus-gene-0.7-mRNA-1	AT3G48110.1	AT3G48110.1	0.09	0.00	0.00	0.13
scaffold07739	maker-scaffold07739-augustus-gene-0.7-mRNA-2	AT3G48110.1	AT3G48110.1	0.09	0.00	0.00	0.13
scaffold10721	maker-scaffold10721-snap-gene-0.12-mRNA-1	AT3G13080.1; AT3G60160.1; AT1G04120.1; AT3G60970.1	AT3G60160.1	0.00	0.00	0.00	0.17
scaffold10655	snap_masked-scaffold10655-abinit-gene-0.6-mRNA-1	-	AT2G44930.1	−0.06	0.41	2.27	0.34
scaffold18444	snap_masked-scaffold18444-abinit-gene-0.3-mRNA-1	-	AT3G14470.1	−0.10	-0.09	0.16	0.35

## Supplementary Materials

The following are available online at https://www.mdpi.com/2223-7747/9/8/1048/s1, Figure S1: Detailed genetic linkage maps of *Castanea* spp. ‘Bouche de Bétizac’ (Bouche) and ‘Madonna’ (Madonna) cultivars. Bouche (female parent, LGs on the left) and Madonna (male parent, LGs on the right). Homologues LGs are presented side-by-side and aligned on the base of markers developed on common scaffolds, here connected with a line. “Ref” indicates *C. mollissima* consensus map, constructed by Kubisiak et al. [18]. The SSRs denoted by ‘CmSIxxxx’ and the SNPs on the same scaffold were used to anchor these maps, Figure S2: Modules used for infestation trials. The screenhouses used to isolate chestnut progeny from the external environment consisted of a metal structure with an anti-aphid net, Table S1: List of SSR markers used in the present study. A complete list of SSR markers used to construct ‘Bouche de Bétizac’ (Bouche) and ‘Madonna’ (Madonna) linkage maps and their publication origins, Table S2: Segregation of resistance to *Dryocosmus kuriphilus*, Table S3: List of SNPs and indels used in the present study. A complete list of SNP and indel markers used to construct ‘Bouche de Bétizac’ (Bouche) and ‘Madonna’ (Madonna) linkage maps and their scaffold (bp) and linkage group position (cM), File S1: Samtools-based pipeline. Bash script containing all the commands used in the Samtools-based pipeline adopted for SNP and indel mining.

## References

[1] Bounous G., Marinoni D.T. (2010). Chestnut: Botany, Horticulture, and Utilization. Hortic. Rev..

[2] Pereira-Lorenzo S., Ballester A., Corredoira E., Viéitez A.M., Agnanostakis S., Costa R.L., Bounous G., Botta R., Beccaro G.L., Kubisiak T.L., Badenes M.L., Byrne D.H. (2012). Chestnut. Fruit Breeding.

[3] Torello-Marinoni D., Nishio S., Portis E., Valentini N., Sartor C., Dini F., Ruffa P., Oglietti S., Martino G., Akkak A. (2018). Development of a genetic linkage map for molecular breeding of chestnut. Acta Hortic..

[4] Santos C., Nelson C.D., Zhebentyayeva T., Machado H., Gomes-Laranjo J., Costa R.L. (2017). First interspecific genetic linkage map for *Castanea sativa* × *Castanea crenata* revealed QTLs for resistance to *Phytophthora cinnamomi*. PLoS ONE.

[5] Santos C., Zhebentyayeva T., Serrazina S., Nelson C.D., Costa R.L. (2015). Development and characterization of EST-SSR markers for mapping reaction to *Phytophthora cinnamomi* in *Castanea* spp.. Sci. Hortic..

[6] Hebard F.V. (2006). The backcross breeding program of the American Chestnut Foundation. J. Am. Chestnut Found.

[7] Tanaka K., Kotobuki K. (1992). Comparative Ease of Pellicle Removal among Japanese Chestnut (*Castanea crenata* Sieb. et Zucc.) and Chinese Chestnut (*C. mollissima Blume*) and Their Hybrids. J. Jpn. Soc. Hortic. Sci..

[8] Shimura I. (2003). Chestnut breeding history. Agric. Hortic..

[9] Moriya S., Inoue K., Otake A., Shiga M., Mabuchi M. (1989). Decline of the chestnut gallwasp population, *Dryocosmus kuriphilus* Yasumatsu (*Hymenoptera*: Cynipidae) after the establishment of *Torymus sinensis* Kajimo (*Hymenoptera*: Torymidae). Appl. Entomol. Zool..

[10] Aebi A., Schönrogge K., Melika G., Alma A., Bosio G., Quacchia A., Picciau L., Abe Y., Moriya S., Yara K., Ozaki K., Yukwa J., Ohgushi T., Price P.W. (2007). Parasitoid Recruitment to the Globally Invasive Chestnut Gall Wasp Dryocosmus kuriphilus. Galling Arthropods and Their Associates.

[11] Sartor C., Dini F., Marinoni D.T., Mellano M.G., Beccaro G.L., Alma A., Quacchia A., Botta R. (2015). Impact of the Asian wasp *Dryocosmus kuriphilus* (Yasumatsu) on cultivated chestnut: Yield loss and cultivar susceptibility. Sci. Hortic..

[12] Dini F., Sartor C., Botta R. (2012). Detection of a hypersensitive reaction in the chestnut hybrid ‘Bouche de Bétizac’ infested by *Dryocosmus kuriphilus* Yasumatsu. Plant Physiol. Biochem..

[13] Kubisiak T.L., Hebard F.V., Nelson C.D., Zhang J., Bernatzky R., Huang H., Anagnostakis S.L., Doudrick R.L. (1997). Molecular Mapping of Resistance to Blight in an Interspecific Cross in the genus Castanea. Phytopathology.

[14] Barreneche T., Casasoli M., Russell K., Akkak A., Meddour H., Plomion C., Villani F., Kremer A. (2003). Comparative mapping between *Quercus* and *Castanea* using simple-sequence repeats (SSRs). Theor. Appl. Genet..

[15] Casasoli M., Mattioni C., Cherubini M., Villani F. (2001). A genetic linkage map of European chestnut (*Castanea sativa* Mill.) based on RAPD, ISSR and isozyme markers. Theor. Appl. Genet..

[16] Casasoli M., Derory J., Morera-Dutrey C., Brendel O., Porth I., Guehl J.-M., Villani F., Kremer A. (2005). Comparison of Quantitative Trait Loci for Adaptive Traits Between Oak and Chestnut Based on an Expressed Sequence Tag Consensus Map. Genetics.

[17] HGW Hardwood Genomic Project. https://hardwoodgenomics.org/Genome-assembly/1962958.

[18] Kubisiak T.L., Nelson C.D., Staton M.E., Zhebentyayeva T., Smith C., Olukolu B.A., Fang G.-C., Hebard F.V., Anagnostakis S., Wheeler N. (2012). A transcriptome-based genetic map of Chinese chestnut (*Castanea mollissima*) and identification of regions of segmental homology with peach (*Prunus persica*). Tree Genet. Genomes.

[19] Nishio S., Takada N., Yamamoto T., Terakami S., Hayashi T., Sawamura Y., Saito T. (2013). Mapping and pedigree analysis of the gene that controls the easy peel pellicle trait in Japanese chestnut (*Castanea crenata* Sieb. et Zucc.). Tree Genet. Genomes.

[20] Nishio S., Terakami S., Matsumoto T., Yamamoto T., Takada N., Kato H., Katayose Y., Saito T. (2018). Identification of QTLs for Agronomic Traits in the Japanese Chestnut (*Castanea crenata* Sieb. et Zucc.) Breeding. Hortic. J..

[21] Casasoli M., Pot D., Plomion C., Monteverdi M.C., Barreneche T., Lauteri M., Villani F. (2004). Identification of QTLs affecting adaptive traits in *Castanea sativa* Mill. Plant Cell Environ..

[22] Elshire R.J., Glaubitz J.C., Sun Q., Poland J., Kawamoto K., Buckler E.S., Mitchell S.E. (2011). A Robust, Simple Genotyping-by-Sequencing (GBS) Approach for High Diversity Species. PLoS ONE.

[23] Peterson B.K., Weber J.N., Kay E.H., Fisher H.S., Hoekstra H.E. (2012). Double Digest RADseq: An Inexpensive Method for De Novo SNP Discovery and Genotyping in Model and Non-Model Species. PLoS ONE.

[24] Shirasawa K., Hirakawa H., Isobe S. (2016). Analytical workflow of double-digest restriction site-associated DNA sequencing based on empirical andin silicooptimization in tomato. DNA Res..

[25] Van Dijk E.L., Auger H., Jaszczyszyn Y., Thermes C. (2014). Ten years of next-generation sequencing technology. Trends Genet..

[26] Bai B., Wang L., Lee M., Zhang Y., Alfiko Y., Ye B.Q., Wan Z.Y., Lim C.H., Suwanto A., Chua N.-H. (2017). Genome-wide identification of markers for selecting higher oil content in oil palm. BMC Plant Biol..

[27] Nagano S., Shirasawa K., Hirakawa H., Maeda F., Ishikawa M., Isobe S.N. (2017). Discrimination of candidate subgenome-specific loci by linkage map construction with an S1 population of octoploid strawberry (*Fragaria* × *Ananassa*). BMC Genom..

[28] Yagi M., Shirasawa K., Waki T., Kume T., Isobe S., Tanase K., Yamaguchi H. (2017). Construction of an SSR and RAD marker-based benetic linkage map for Carnation (*Dianthus caryophyllus* L.). Plant Mol. Biol. Rep..

[29] Zhong Y.-J., Zhou Y.-Y., Li J.-X., Yu T., Wu T.-Q., Luo J.-N., Luo S.-B., Huang H. (2017). A high-density linkage map and QTL mapping of fruit-related traits in pumpkin (*Cucurbita moschata* Duch.). Sci. Rep..

[30] UNIPROT. https://www.uniprot.org/uniprot/Q9LRR4.

[31] Acquadro A., Marinoni D.T., Sartor C., Dini F., Macchio M., Botta R. (2019). Transcriptome characterization and expression profiling in chestnut cultivars resistant or susceptible to the gall wasp *Dryocosmus kuriphilus*. Mol. Genet. Genom..

[32] Bonierbale M.W., Plaisted R.L., Tanksley S.D. (1988). RFLP Maps Based on a Common Set of Clones Reveal Modes of Chromosomal Evolution in Potato and Tomato. Genetics.

[33] Causse M.A., Fulton T.M., Cho Y.G., Ahn S.N., Chunwongse J., Wu K., Xiao J., Yu Z., Ronald P.C., Harrington S.E. (1994). Saturated Molecular Map of the Rice Genome Based on an Interspecific Backcross Population. Genetics.

[34] Gebhardt C., Ritter E., Barone A., Debener T., Walkemeier B., Schachtschabel U., Kaufmann H., Thompson R.D., Bonierbale M.W., Ganal M.W. (1991). FFLP maps of potato and their alignment with the homologous tomato genome. Theor. Appl. Genet..

[35] Ky C.L., Barre P., Lorieux M., Trouslot P., Akaffou S., Louarn J., Charrier A., Hamon S., Noirot M. (2000). Interspecific genetic linkage map, segregation distortion and genetic conversion in coffee (*Coffea* sp.). Theor. Appl. Genet..

[36] Lawrence E.J., Griffin C.H., Henderson I.R. (2017). Modification of meiotic recombination by natural variation in plants. J. Exp. Bot..

[37] Coll N.S., Vercammen D., Smidler A., Clover C., Van Breusegem F., Dangl J.L., Epple P. (2010). Arabidopsis Type I Metacaspases Control Cell Death. Science.

[38] Bittner-Eddy P.D., Beynon J. (2001). The Arabidopsis Downy Mildew Resistance Gene, RPP13-Nd, Functions Independently of NDR1 and EDS1 and Does Not Require the Accumulation of Salicylic Acid. Mol. Plant Microbe Interact..

[39] Ferracini C., Gonella E., Ferrari E., Saladini M.A., Picciau L., Tota F., Pontini M., Alma A. (2014). Novel insight in the life cycle of *Torymus sinensis*, biocontrol agent of the chestnut gall wasp. BioControl.

[40] Kotobuki K., Saito T., Kashimura Y., Shoda M. (1999). Chestnut breeding program in national institute of fruit tree science, Japan. Acta Hortic..

[41] Doyle J.J., Doyle J.L. (1987). A rapid DNA isolation procedure from small quantities of fresh leaf tissue. Phytochem. Bull..

[42] Aldrich P.R., Michler C.H., Sun W., Romero-Severson J. (2002). Microsatellite markers for northern red oak (Fagaceae: *Quercus rubra*). Mol. Ecol. Notes.

[43] Buck E., Hadonou M., James C.J., Blakesley D., Russell K. (2003). Isolation and characterization of polymorphic microsatellites in European chestnut (*Castanea sativa* Mill.). Mol. Ecol. Notes.

[44] Kampfer S., Lexer C., Glössl J., Steinkellner H. (2004). Characterization of (GA) n Microsatellite Loci from *Quercus Robur*. Hereditas.

[45] Marinoni D., Akkak A., Bounous G., Edwards K.J., Botta R. (2003). Development and characterization of microsatellite markers in *Castanea sativa* (Mill.). Mol. Breed..

[46] Nishio S., Yamamoto T., Terakami S., Sawamura Y., Takada N., Nishitani C., Saito T. (2011). Novel genomic and EST-derived SSR markers in Japanese chestnuts. Sci. Hortic..

[47] Steinkellner H., Fluch S., Turetschek E., Lexer C., Streiff R., Kremer A., Burg K., Glössl J. (1997). Identification and characterization of (GA/CT) n-microsatellite loci from *Quercus petraea*. Plant Mol. Biol..

[48] Acquadro A., Barchi L., Gramazio P., Portis E., Vilanova S., Comino C., Plazas M., Prohens J., Lanteri S. (2017). Coding SNPs analysis highlights genetic relationships and evolution pattern in eggplant complexes. PLoS ONE.

[49] Li H., Durbin R. (2009). Fast and accurate short read alignment with Burrows-Wheeler transform. Bioinformatics.

[50] Li H., Handsaker B., Wysoker A., Fennell T., Ruan J., Homer N., Marth G., Abecasis G.R., Durbin R. (2009). The Sequence Alignment/Map format and SAMtools. Bioinformatics.

[51] Van Ooijen J. (2006). JoinMap^®^ 4, Software for the Calculation of Genetic Linkage Maps in Experimental Populations.

[52] Grattapaglia D., Sederoff R. (1994). Genetic Linkage Maps of *Eucalyptus Grandis* and *Eucalyptus Urophylla* using a pseudo-testcross: Mapping Strategy and Rapd Markers. Genetics.

[53] Kosambi D.D. (1943). The estimation of map distances from recombination values. Ann. Eugen..

[54] Voorrips R.E. (2002). MapChart: Software for the graphical presentation of linkage maps and QTLs. J. Hered..

[55] Lander E.S., Botstein D. (1989). Mapping Mendelian Factors Underlying Quantitative Traits Using RFLP Linkage Maps. Genetics.

[56] Jansen R.C., Stam P. (1994). High resolution of quantitative traits into multiple loci via interval mapping. Genetics.

[57] Van Ooijen J.W., Boer M.P., Jansen R.C., Maliepaard C. (2002). MapQTL 4.0: Software for the Calculation of QTL Positions on Genetic Maps.

[58] Churchill G.A., Doerge R.W. (1994). Empirical Threshold Values for Quantitative Trait Mapping. Genetics.

