# Supplementary material for: Development of High-Density Genetic Linkage Maps and Identification of Loci for Chestnut Gall Wasp Resistance in Castanea spp
Source: Plants (Basel). 2020 Aug 18;9(8):1048. doi: 10.3390/plants9081048 (PMC7465717; doi:10.3390/plants9081048)
Supplement: Supplementary file 1 [file plants-09-01048-s001.zip › Supplementary material_Table S1_Torello Marinoni and NIshio et al_revised manuscript_ROUND 2.docx]

**Supplementary material**

**Development of high-density genetic linkage maps and identification of loci for chestnut gall wasp resistance in *Castanea* spp.**

Daniela Torello Marinoni^1#^, Sogo Nishio^2#^, Nadia Valentini^1^, Kenta Shirasawa^3^, Alberto Acquadro^1^, Ezio Portis^1^, Alberto Alma^1^, Aziz Akkak^4^, Vera Pavese^1^, Emile Cavalet Giorsa^1^, Roberto Botta^1^

^#^ These authors contributed equally to this work.

1 Dipartimento di Scienze Agrarie, Forestali e Alimentari - DISAFA, Università degli Studi di Torino, Largo Paolo Braccini 2, 10095 Grugliasco, (TO), Italy

2 Institute of Fruit Tree and Tea Science, NARO, 2-1 Fujimoto, Tsukuba, Ibaraki 305-8605, Japan

3 Kazusa DNA Research Institute, 2-6-7 Kazusa-Kamatari, Kisarazu, Chiba 292-0818, Japan

4 Dipartimento di Scienze Agrarie, degli Alimenti e dell'Ambiente, Università degli Studi di Foggia, Via Napoli 25, 71121 Foggia, Italy

corresponding author:

Daniela Torello Marinoni

[daniela.marinoni@unito.it](mailto:daniela.marinoni@unito.it)

phone number +390116708816

fax numbers +0390116708658

ORCID: [orcid.org/0000-0002-3679-4813](https://orcid.org/0000-0002-3679-4813)

**Supplementary material**

Table S1**. List of SSR markers used in the present study.** A complete list of SSR markers used to construct **‘**Bouche de Bétizac’ (Bouche) and ‘Madonna’ (Madonna) linkage maps and their publication origins.

| Marker | Origin | Reference |
| --- | --- | --- |
| 10_145 | *C. sativa x C. crenata* | Akkak , personal communication |
| 21_145 | *C. sativa x C. crenata* | Akkak , Personal communication |
| 22_145 | *C. sativa x C. crenata* | Akkak , Personal communication |
| 3_145 | *C. sativa x C. crenata* | Akkak , Personal communication |
| 4_145 | *C. sativa x C. crenata* | Akkak , Personal communication |
| 7_145 | *C. sativa x C. crenata* | Akkak , Personal communication |
| CmSI0009 | *C. mollissima* | Kubisiak et al. 2013 |
| CmSI0013 | *C. mollissima* | Kubisiak et al. 2013 |
| CmSI0045 | *C. mollissima* | Kubisiak et al. 2013 |
| CmSI0071 | *C. mollissima* | Kubisiak et al. 2013 |
| CmSI0247 | *C. mollissima* | Kubisiak et al. 2013 |
| CmSI0249 | *C. mollissima* | Kubisiak et al. 2013 |
| CmSI0262 | *C. mollissima* | Kubisiak et al. 2013 |
| CmSI0327 | *C. mollissima* | Kubisiak et al. 2013 |
| CmSI0385 | *C. mollissima* | Kubisiak et al. 2013 |
| CmSI0396 | *C. mollissima* | Kubisiak et al. 2013 |
| CmSI0401 | *C. mollissima* | Kubisiak et al. 2013 |
| CmSI0404 | *C. mollissima* | Kubisiak et al. 2013 |
| CmSI0407 | *C. mollissima* | Kubisiak et al. 2013 |
| CmSI0430 | *C. mollissima* | Kubisiak et al. 2013 |
| CmSI0437 | *C. mollissima* | Kubisiak et al. 2013 |
| CmSI0513 | *C. mollissima* | Kubisiak et al. 2013 |
| CmSI0516 | *C. mollissima* | Kubisiak et al. 2013 |
| CmSI0523 | *C. mollissima* | Kubisiak et al. 2013 |
| CmSI0531 | *C. mollissima* | Kubisiak et al. 2013 |
| CmSI0550 | *C. mollissima* | Kubisiak et al. 2013 |
| CmSI0555 | *C. mollissima* | Kubisiak et al. 2013 |
| CmSI0556 | *C. mollissima* | Kubisiak et al. 2013 |
| CmSI0561 | *C. mollissima* | Kubisiak et al. 2013 |
| CmSI0562 | *C. mollissima* | Kubisiak et al. 2013 |
| CmSI0585 | *C. mollissima* | Kubisiak et al. 2013 |
| CmSI0611 | *C. mollissima* | Kubisiak et al. 2013 |
| CmSI0617 | *C. mollissima* | Kubisiak et al. 2013 |
| CmSI0630 | *C. mollissima* | Kubisiak et al. 2013 |
| CmSI0632 | *C. mollissima* | Kubisiak et al. 2013 |
| CmSI0644 | *C. mollissima* | Kubisiak et al. 2013 |
| CmSI0668 | *C. mollissima* | Kubisiak et al. 2013 |
| CmSI0689 | *C. mollissima* | Kubisiak et al. 2013 |
| CmSI0691 | *C. mollissima* | Kubisiak et al. 2013 |
| CmSI0702 | *C. mollissima* | Kubisiak et al. 2013 |
| CmSI0707 | *C. mollissima* | Kubisiak et al. 2013 |
| CmSI0727 | *C. mollissima* | Kubisiak et al. 2013 |
| CmSI0740 | *C. mollissima* | Kubisiak et al. 2013 |
| CmSI0741 | *C. mollissima* | Kubisiak et al. 2013 |
| CmSI0744 | *C. mollissima* | Kubisiak et al. 2013 |
| CmSI0745 | *C. mollissima* | Kubisiak et al. 2013 |
| CmSI0747 | *C. mollissima* | Kubisiak et al. 2013 |
| CmSI0749 | *C. mollissima* | Kubisiak et al. 2013 |
| CmSI0751 | *C. mollissima* | Kubisiak et al. 2013 |
| CmSI0752 | *C. mollissima* | Kubisiak et al. 2013 |
| CmSI0760 | *C. mollissima* | Kubisiak et al. 2013 |
| CmSI0785 | *C. mollissima* | Kubisiak et al. 2013 |
| CmSI0800 | *C. mollissima* | Kubisiak et al. 2013 |
| CmSI0804 | *C. mollissima* | Kubisiak et al. 2013 |
| CmSI0815 | *C. mollissima* | Kubisiak et al. 2013 |
| CmSI0849 | *C. mollissima* | Kubisiak et al. 2013 |
| CmSI0875 | *C. mollissima* | Kubisiak et al. 2013 |
| CmSI0894 | *C. mollissima* | Kubisiak et al. 2013 |
| CmSI0911 | *C. mollissima* | Kubisiak et al. 2013 |
| CmSI0914 | *C. mollissima* | Kubisiak et al. 2013 |
| CmSI0921 | *C. mollissima* | Kubisiak et al. 2013 |
| CmSI0922 | *C. mollissima* | Kubisiak et al. 2013 |
| CmSI0925 | *C. mollissima* | Kubisiak et al. 2013 |
| CmSI0931 | *C. mollissima* | Kubisiak et al. 2013 |
| CmSI0934 | *C. mollissima* | Kubisiak et al. 2013 |
| CmSI0935 | *C. mollissima* | Kubisiak et al. 2013 |
| CmSI0945 | *C. mollissima* | Kubisiak et al. 2013 |
| CONS-58 | *C. sativa* | Marinoni et al. 2013 |
| CsCAT1 | *C. sativa* | Marinoni et al. 2013 |
| CsCAT14 | *C. sativa* | Marinoni et al. 2013 |
| CsCAT15 | *C. sativa* | Marinoni et al. 2013 |
| CsCAT17 | *C. sativa* | Marinoni et al. 2013 |
| CsCAT3 | *C. sativa* | Marinoni et al. 2013 |
| CsCAT34 | *C. sativa* | Marinoni et al. 2013 |
| CsCAT6 | *C. sativa* | Marinoni et al. 2013 |
| CsCAT7 | *C. sativa* | Marinoni et al. 2013 |
| CsCAT8 | *C. sativa* | Marinoni et al. 2013 |
| EMCs11 | *C. sativa* | Buck et al. 2013 |
| EMCs2 | *C. sativa* | Buck et al. 2013 |
| EMCs22 | *C. sativa* | Buck et al. 2013 |
| EMCs32 | *C. sativa* | Buck et al. 2013 |
| EMCs4 | *C. sativa* | Buck et al. 2013 |
| PEA100 | *C. crenata* | Nishio et al. 2011 |
| PEA111 | *C. crenata* | Nishio et al. 2011 |
| PEA2 | *C. crenata* | Nishio et al. 2011 |
| PEA50 | *C. crenata* | Nishio et al. 2011 |
| PEA8 | *C. crenata* | Nishio et al. 2011 |
| PEA83 | *C. crenata* | Nishio et al. 2011 |
| PEA89 | *C. crenata* | Nishio et al. 2011 |
| PEB100 | *C. crenata* | Nishio et al. 2011 |
| PEB102 | *C. crenata* | Nishio et al. 2011 |
| PEB119 | *C. crenata* | Nishio et al. 2011 |
| PEB32 | *C. crenata* | Nishio et al. 2011 |
| PEB7 | *C. crenata* | Nishio et al. 2011 |
| PRA25 | *C. crenata* | Nishio et al. 2011 |
| PRA76 | *C. crenata* | Nishio et al. 2011 |
| PRB9 | *C. crenata* | Nishio et al. 2011 |
| PRC24 | *C. crenata* | Nishio et al. 2011 |
| PRC4 | *C. crenata* | Nishio et al. 2011 |
| PRC9 | *C. crenata* | Nishio et al. 2011 |
| PRD105 | *C. crenata* | Nishio et al. 2011 |
| PRD31 | *C. crenata* | Nishio et al. 2011 |
| PRD55 | *C. crenata* | Nishio et al. 2011 |
| PRD58 | *C. crenata* | Nishio et al. 2011 |
| PRD66 | *C. crenata* | Nishio et al. 2011 |
| PRD67 | *C. crenata* | Nishio et al. 2011 |
| PRD82 | *C. crenata* | Nishio et al. 2011 |
| PRD96 | *C. crenata* | Nishio et al. 2011 |
| PRF106 | *C. crenata* | Nishio et al. 2011 |
| PRF41 | *C. crenata* | Nishio et al. 2011 |
| PRM5 | *C. crenata* | Nishio et al. 2011 |
| QpZAG110 | *Quercus petraea* | Steinkellne et al. 1997 |
| QpZAG36 | *Quercus petraea* | Steinkellne et al. 1997 |
| QrZAG20 | *Quercus rubra* | Kampfer et al. 1998 |
| quru-GA-0C11 | *Quercus rubra* | Aldrich et al. 2002 |
| UFCS12 | *C. sativa x C. crenata* | Akkak , Personal communication |
| UFCS15 | *C. sativa x C. crenata* | Akkak , Personal communication |
| UFCS18 | *C. sativa x C. crenata* | Akkak , Personal communication |
| UFCS23 | *C. sativa x C. crenata* | Akkak , Personal communication |
| UFCS24 | *C. sativa x C. crenata* | Akkak , Personal communication |
| UFCS25 | *C. sativa x C. crenata* | Akkak , Personal communication |
| UFCS31 | *C. sativa x C. crenata* | Akkak , Personal communication |
| UFCS37 | *C. sativa x C. crenata* | Akkak , Personal communication |
| UFCS40 | *C. sativa x C. crenata* | Akkak , Personal communication |
| UFCS50 | *C. sativa x C. crenata* | Akkak , Personal communication |
| UFCS53 | *C. sativa x C. crenata* | Akkak , Personal communication |
| UFCS54 | *C. sativa x C. crenata* | Akkak , Personal communication |
| UFCS56 | *C. sativa x C. crenata* | Akkak , Personal communication |
| UFCS60 | *C. sativa x C. crenata* | Akkak , Personal communication |
| UFCS63 | *C. sativa x C. crenata* | Akkak , Personal communication |
