# Supplementary figures and images for: Development of High-Density Genetic Linkage Maps and Identification of Loci for Chestnut Gall Wasp Resistance in Castanea spp
Source: Plants (Basel). 2020 Aug 18;9(8):1048. doi: 10.3390/plants9081048 (PMC7465717; doi:10.3390/plants9081048)

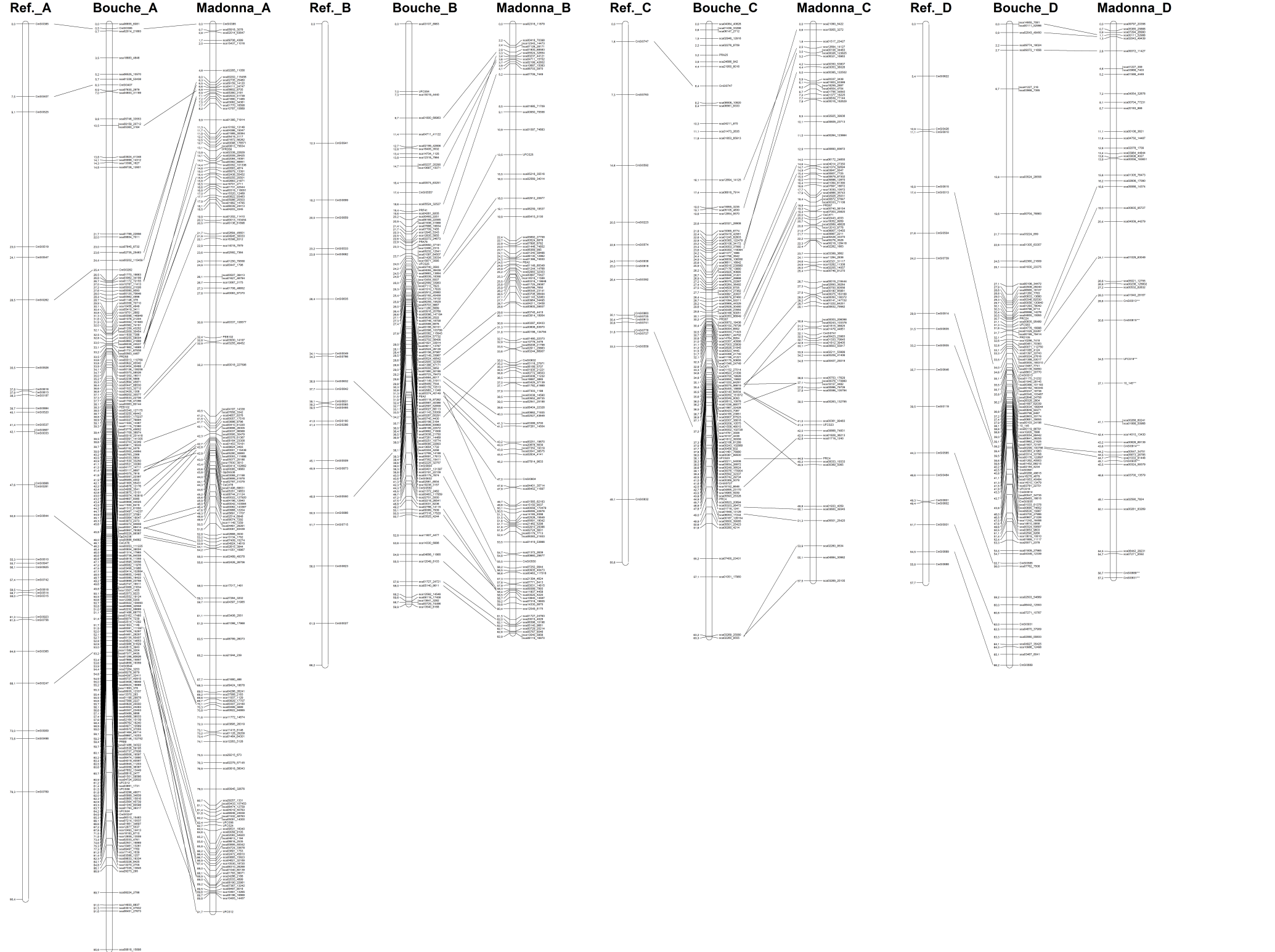

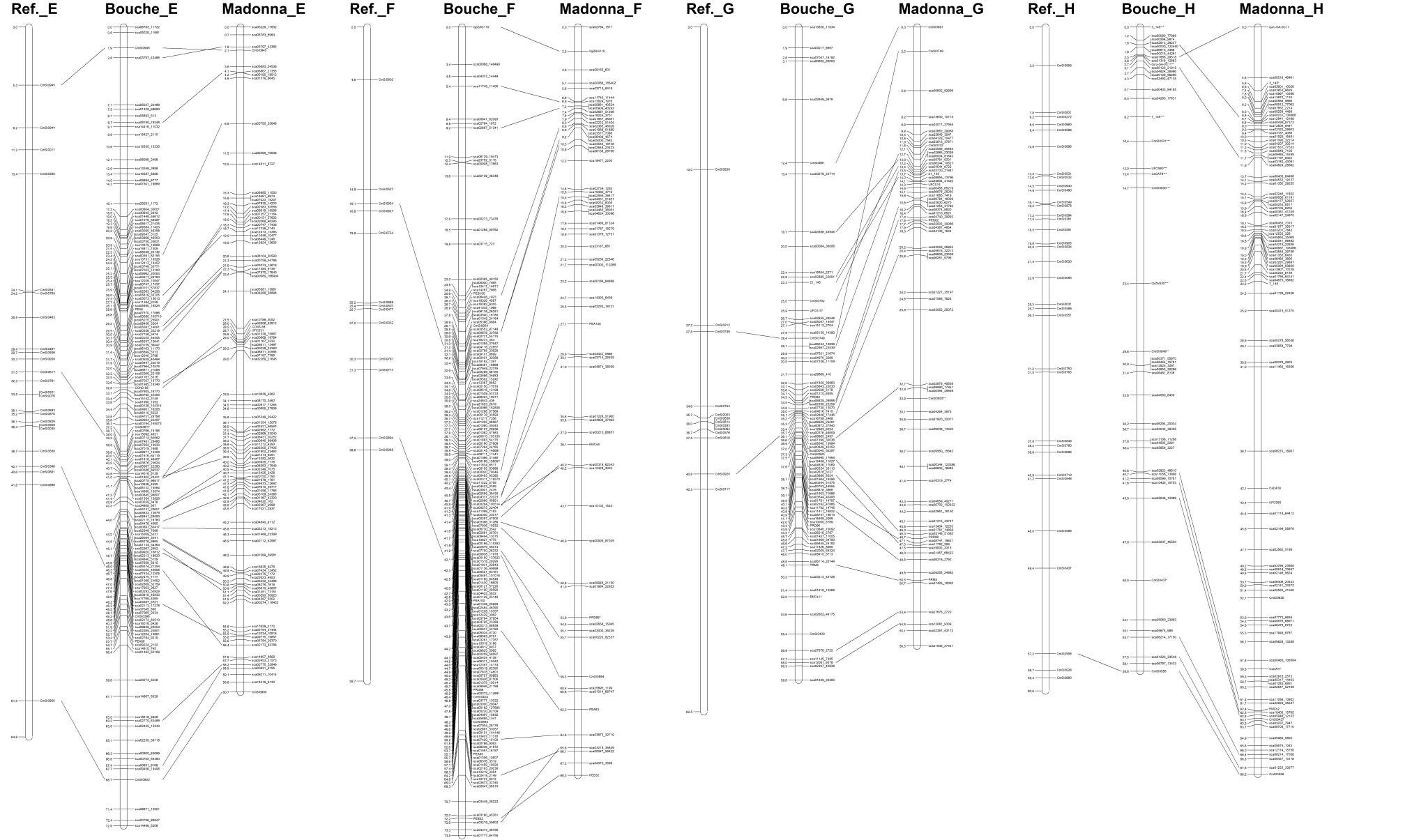

Supplement: Supplementary file 1 [file plants-09-01048-s001.zip › Supplementary material_Figure S1_Torello Marinoni and Nishio et al_revised manuscript_ROUND 2.pdf]

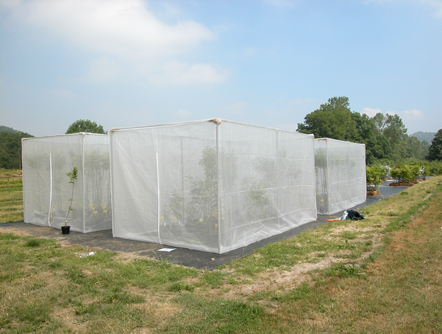

Supplement: Supplementary file 1 [file plants-09-01048-s001.zip › Supplementary material_Figure S2_Torello Marinoni and Nishio et al_revised manuscript_ROUND 2.bmp]
